# Exposure to electronic cigarette vapors affects pulmonary and systemic expression of circadian molecular clock genes

**DOI:** 10.14814/phy2.13440

**Published:** 2017-10-16

**Authors:** Ariane Lechasseur, Éric Jubinville, Joanie Routhier, Jean‐Christophe Bérubé, Mélanie Hamel‐Auger, Maude Talbot, Jennifer Lamothe, Sophie Aubin, Marie‐Ève Paré, Marie‐Josée Beaulieu, Yohan Bossé, Caroline Duchaine, Mathieu C. Morissette

**Affiliations:** ^1^ Quebec Heart and Lung Institute ‐ Université Laval Quebec City Province of Quebec Canada; ^2^ Faculty of Medicine Université Laval Quebec City Province of Quebec Canada; ^3^ Department of Molecular Medicine Faculty of Medicine Université Laval Quebec City Province of Quebec Canada; ^4^ Department of Biochemistry, Microbiology and Bioinformatics Faculty of Sciences and Engineering Quebec City Province of Quebec Canada; ^5^ Department of Medicine Faculty of Medicine Université Laval Quebec City Province of Quebec Canada

**Keywords:** Animal model, circadian molecular clock, electronic cigarette, Glycerol, lungs, Propylene glycol

## Abstract

E‐cigarette use has exploded in the past years, especially among young adults and smokers desiring to quit. While concerns are mostly based on the presence of nicotine and flavors, pulmonary effects of propylene glycol and glycerol inhalation, the main solvents of e‐liquid have not been thoroughly investigated. In this preclinical study, mice were exposed 2 h daily for up to 8 weeks to vapors of propylene glycol and/or glycerol generated by an e‐cigarette. Lung transcriptome analysis revealed it affected the expression level of genes of the circadian molecular clock, despite causing no inflammatory response. Periodical sacrifices showed that the rhythmicity of these regulatory genes was indeed altered in the lungs, but also in the liver, kidney, skeletal muscle, and brain. E‐cigarette exposure also altered the expression of rhythmic genes (i.e., *hspa1a* and *hspa1b*), suggesting that alterations to the ‘clock genes’ could translate into systemic biological alterations. This study reveals that the major solvents used in e‐cigarettes propylene glycol and glycerol, not nicotine or flavors, have unsuspected effects on gene expression of the molecular clock that are to be taken seriously, especially considering the fundamental role of the circadian rhythm in health and disease.

## Introduction

Electronic cigarette use, or vaping, is becoming more and more popular worldwide. In fact, a collection of studies and surveys has shown that a significant proportion of teenagers and adults has tried to or is vaping, of which most are current or former tobacco users (Etter and Bullen 2014; Camenga et al. 2015; Farsalinos et al. 2015; Reid et al. 2015; Shiplo et al. 2015). Despite the aura of safety surrounding vaping, reality is that we currently know very little about its pulmonary and systemic effects. This is especially true for the consequences of chronic inhalation of e‐liquid solvents, such as propylene glycol and glycerol, representing most of the e‐liquid volume (Grana et al. 2014; Flora et al. 2016). Therefore, as matter of public safety and risk assessment, we need to better understand the impact of these two compounds when inhaled on a chronic basis using an electronic cigarette device.

Propylene glycol (C_3_H_8_O_2_) is a synthetic compound and is used in numerous applications from food processing to polymer synthesis (West et al. 2014). Glycerol (C_3_H_8_O_3_), or vegetable glycerine, is a sweet‐tasting compound that is endogenously produced in living organisms and widely used in the food industry (Robergs and Griffin 1998). The oral toxicity of propylene glycol and glycerol is very low, an argument often used to promote the safety of vaping. Whereas the digestive tract can easily process these compounds, their inhalation using an electronic cigarette device may affect the respiratory system very differently. In fact, the lungs are not used to be exposed to such a massive amount of propylene glycol or glycerol on a chronic basis. This leaves the possibility for unwanted or even health‐threatening biological processes to be triggered.

Research shows that vaping is unlikely to have noticeable acute pulmonary effects, this new social habit being too recent for retrospective epidemiological studies to assess long‐term effects (Flouris et al. 2013; Lerner et al. 2016; Larcombe et al. 2017). Studies using animal modeling of chronic exposure to electronic cigarette vapors have shown potential adverse pulmonary effects, including signs of airspace enlargement and altered antimicrobial defense (Sussan et al. 2015; Garcia‐Arcos et al. 2016; Hwang J. H. et al. 2016). However, these phenomena were largely attributable to the nicotine contained in the e‐liquid and do not extensively document the possible impact of propylene glycol and glycerol alone outside of the ‘standard’ tobacco‐related outcomes.

To specifically dissect the impact of repeated propylene glycol and glycerol inhalation using an electronic cigarette device, we exposed mice to vapors of nicotine‐free and flavor‐free propylene glycol and glycerol generated using a commercially available electronic cigarette for up to 8 weeks. Using microarray lung transcriptomics analysis, we found that exposure to electronic cigarette‐generated vapors of propylene glycol and glycerol, in combination or separately, caused pulmonary transcriptional alterations in genes of the circadian molecular clock. Surprisingly, these effects were not restricted to the lungs but extended to the liver, brain, skeletal muscle, and kidney. Knowing the importance of the circadian molecular clock in biological homeostasis and diseases, these alterations could potentially facilitate the onset of adverse health conditions, including lung and metabolic chronic diseases, infections, and cancer.

## Methods

### Mice and electronic cigarette vapors exposure

**Figure 1:**
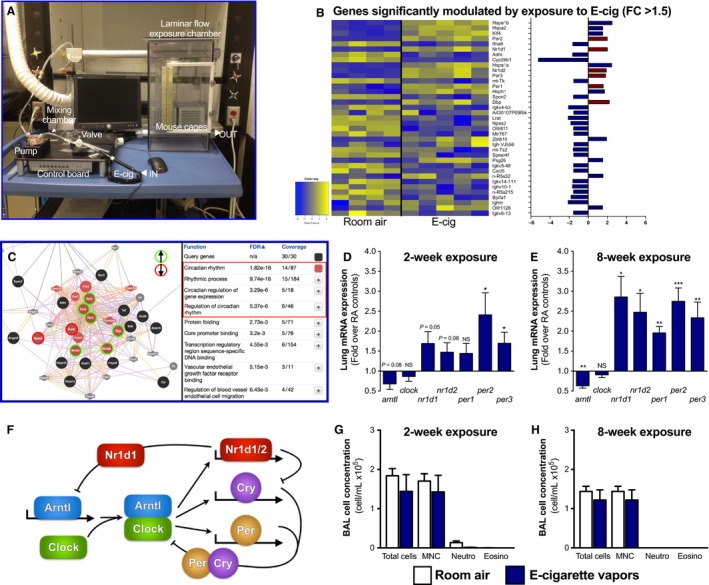
Exposure to electronic cigarette vapors of propylene glycol and glycerol affects the expression of circadian molecular clock genes in the lungs despite causing no inflammation. (A) Automated mouse whole‐body exposure system to electronic cigarette vapors. (B) Microarray data showing genes significantly modulated in the lungs (*P *< 0.05; fold change >1.5) following an 8‐week exposure to propylene glycol 70%/glycerol 30% vapors from electronic cigarette (BALB/c; *n* = 5/group). (C) Assessment of the functional relationship between the genes modulated by electronic cigarette exposure in the lungs using the Genemania platform. (D–E) Quantitative PCR assessment of the key circadian genes in lungs from mice exposure for 2 weeks and 8 weeks to propylene glycol 70%/glycerol 30% vapors from electronic cigarette. (F) Schematic representation of the CLOCK system, which controls circadian rhythmicity. BAL total and differential cell counts from mice exposed to room air (open bars) or e‐cigarette vapors from propylene glycol 70%/glycerol 30% (blue bares) for (G) 2 and (H) 8 weeks. Data are presented as mean ± SEM. Mann–Whitney nonparametric test. NS: nonsignificant; *: *P* < 0.05; **: *P* < 0.01; ***: *P* < 0.001.

Six‐ to eight‐week‐old female BALB/c mice were obtained from Charles River (St‐Constant, QC, Canada). Mice were housed in 12:12 light/dark cycles (6 am to 6 pm) with access to food and water ad libitum. Exposure to electronic cigarette vapors took place between 1 pm and 3 pm using our homemade whole‐body exposure system (Fig. 1A). A pump and pinch valve are controlled by a programmable automated system (InExpose control board; SCIREQ Scientific Respiratory Equipment Inc, Montréal, PQ, Canada) to take three 80‐mL puffs per minute from a commercial e‐cigarette. The puffs are then mixed with room air (bias flow of 3L/min) and sent in the whole‐body exposure chamber by laminar flow where mice breathe freely the vapors. The laboratory‐made e‐liquid is typically made of high‐grade USP 70% propylene glycol (PG) and 30% glycerol (Gly), representative of most e‐liquids commercially available. E‐liquids containing 100% PG and 100% Gly were also used with this system. For this study, no nicotine or flavor agents were added to the e‐liquid. Mice were exposed 2 h per day, 5 days a week up to 8 weeks. Control mice were exposed to room air in the same conditions as electronic cigarette‐exposed mice. They were placed next to the exposure system; so they were exposed to the same noise, light intensity, staff presence, etc. No food or water was accessible to either electronic cigarette or room air‐exposed mice during the exposure.

### Periodic sacrifices over 24 h

Female BALB/c mice were exposed for 2 weeks to electronic cigarette vapors from three different e‐liquids containing (1) PG 70%/Gly 30%, (2) PG 100%, or (3) Gly 100%. To ensure that our observations were not caused by environmental differences during the exposure, mice from the control groups were placed in the same room and cages, thereby exposed to same lighting, noises, and vibrations as the mice exposed to electronic cigarette vapors. Starting the day following the last exposure, five mice from the control group and five mice from the exposed group were euthanized every 6 h over 24 h, starting at 6 am (Zeitgeber Time (ZT) 0 = 6 am [lights on]; ZT6 = 12 pm; ZT 12 = 6 pm [lights off]; ZT 18 = 12 am; ZT 24 = 6 am + 1 day). To prevent sudden changes in lighting conditions during sacrifice, mice euthanized during the day were kept in daylight before and during the procedure, and mice euthanized during the night were kept in the dark until fully anesthetized.

### Tissue harvesting

Mice were anesthetized with isoflurane and euthanized by exsanguination by cutting the descending aorta. For periodical sacrifices, tissues were collected sequentially as listed below.

*Lungs*: For periodical sacrifices, lung lobes were detached, snap frozen in liquid nitrogen, and stored at −80°C. For sacrifices with a single time point (data from Fig. 1), lungs were removed from the thoracic cavity and the trachea was cannulated. Right lobes were tied up, dissected, snap frozen in liquid nitrogen, and stored at −80°C for transcriptional analyses. The left lobe was lavaged with 250 and 200 *μ*L of cold PBS sequentially. The lavaged lobe was then inflated with 10% formalin for histological analysis. BAL total cell concentrations were assessed by hemocytometer. Cells were then pelleted (10 min, 800 g, 4°C) and resuspended in PBS for cytospins. Cytospins were stained using Hema3 (Fisher Scientific Company, Ottawa, ON, Canada) for differential counts, for which 300 cells per sample were counted.

*Gastrocnemius (limb muscle)*: Skin from both limbs was removed and both gastrocnemius muscles dissected, snap frozen in liquid nitrogen, and stored at −80°C.

*Brain*: The cranium was exposed and incised from the back. The brain was exposed, collected, snap frozen in liquid nitrogen, and stored at −80°C.

*Liver* and *Kidneys*: Liver and both kidneys were collected, snap frozen in liquid nitrogen, and stored at −80°C.

### Assessment of lung gene expression by microarray

Total RNA was extracted from 30 to 60 mg of frozen lung tissue first using TRIzol reagent (Thermo Fisher Scientific, Waltham, MA) and then RNeasy Plus Universal Mini kit (Qiagen). RNA concentration and purity were assessed by measuring the 260/280 nm ratio using the NanoVue spectrophotometer (GE Healthcare). RNA quality was assessed using the Agilent 2100 Bioanalyzer (Agilent Technologie, CA). Whole‐transcript expression was evaluated using the Affymetrix Mouse Gene 2.0 ST Assay. All gene expression quality controls and analyses were carried out with the R statistical software (R Core Team – 2016. R: A language and environment for statistical computing. R Foundation for Statistical Computing, Vienna, Austria. URL https://www.R-project.org/). Mouse gene expression profiling was measured using the expression GeneChip^®^ Mouse Gene 2.0 ST (Affymetrix) and quality controls were performed with the R package affy. The R package samr and limma were used and genes were considered differentially expressed at a false discovery rate (FDR) < 0.05 and with an absolute fold change > 1.5. T‐tests were also performed to compare the groups. Corrections for multiple comparisons resulted in no significant differences between the groups. Therefore, data from the gene array are presented as uncorrected T‐tests and should be interpreted accordingly. The web‐based freeware GeneMania was then used to predict activated functions and pathways.

### Quantitative PCR

Total RNA was extracted using TRIzol reagent (Thermo Fisher Scientific) and cDNA generated using the iScript Advanced cDNA synthesis kit (Bio‐Rad, Mississauga, ON, Canada). qPCR analyses were performed using SsoAdvanced Universal SYBR Green Supermix (Bio‐Rad) primers at 10 *μ*mol/L. Gene expression levels were assessed using *hprt* and *rplp0* as reference genes using the ∆∆Cq method. All reactions were performed in triplicate. The primers and qPCR conditions used for amplifications were optimized for each gene (see Table 1). qPCR was performed using a CFX384 Touch qPCR System (Bio‐Rad) and data acquired/analyzed with the on‐board CFX Manager software. All qPCR efficiencies were between 90 and 110%, with *R*² values ranging between 0.97 and 1.00. The thermocycle protocol was as follows: 95°C for 3 min, followed by 40 cycles of 95°C for 10 sec, and 57–60°C for 30 sec.

**Table 1:** Primer sequences and annealing temperatures

Gene name	Primer sequences	Annealing temperature (°C)				
		Brain	Kidney	Liver	Lung	Muscle
Arntl	For: CGG TCA CAT CCT ACG ACA AAC Rev: CAG AAG CAA ACT ACA AGC CAA C	57	59	60	57	57
Hprt	For: AGC AGG TCA GCA AAG AAC T Rev: CCT CAT GGA CTG ATT ATG GAC A	57	57	57	57	59
Hspa1a	For: CGA GTT CAG GAT GGT TGT GTA Rev: GGT TGC ACT GTA GGA CTT GT	60	60	60	60	60
Hspa1b	For: GTA GTA CAC AGT GCC AAG ACG Rev: TTT ATA TCA GTG TTC CAG TAG CCT	59	59	59	59	59
Nr1d1	For: GAG CCA CTA GAG CCA ATG TAG Rev: CCA GTT TGA ATG ACC GCT TTC	57	57	57	57	57
Nr1d2	For: ACA GTT CTC ATT CTT CAG GCA Rev: GGC ATC AGG ATT CCA CTA TGG	60	57	57	57	57
Per1	For: CTT TGC TTT AGA TCG GCA GTG Rev: CTT CCT CAA CCG CTT CAG A	57	57	57	57	57
Per2	For: TGA GGT AGA TAG CCC AGG AG Rev: GCT ATG AAG CGC CTA GAA TCC	57	59	57	57	57
Per3	For: CTC TTC TCT CTG TCT CCA CCT Rev: TCC AAC TCA GCT TCC TTT CTG	60	57	60	57	59
Rplp0	For: ATC ACA GAG CAG GCC CTG CA Rev: CAC CGA GGC AAC AGT TGG GT	57	57	57	57	59

### Statistics

Two‐group comparisons were performed using a Mann–Whitney nonparametric test. Corrections for multiple comparisons were performed for both the gene array and the RT‐PCR analyses but no statistical differences were observed. Therefore, data should be interpreted accordingly. Statistical tests were performed using Prism 7 from GraphPad Software, Inc. (La Jolla, CA).

### Study approval

Mice were housed according to the Canadian Council for Animal Care (CCAC) guidelines and Université Laval's Animal Research Ethics Board approved all procedures (Animal utilization protocol #2014121‐2).

## Results

### Electronic cigarette‐mediated exposure to propylene glycol and glycerol vapors rapidly affects the pulmonary expression of circadian molecular clock genes

To investigate the effects of nicotine‐free and flavor‐free electronic cigarette vapors on the lungs, we exposed BALB/c mice to electronic cigarette‐mediated vapors of propylene glycol and glycerol for 2 h/day, 5 days/week for 8 consecutive weeks using our whole‐body exposure system (Fig. 1A). Lungs were harvested and total RNA was extracted to perform transcriptional analyses using expression microarrays. Exposure to electronic cigarette‐mediated vapors of propylene glycol and glycerol significantly modulated the expression of 37 genes in the lungs (fold change > 1.5) (Fig. 1B). Using the Genemania platform (Warde‐Farley et al. 2010), we found the most significant function predicted to be affected by electronic cigarette vapors was the regulation of the circadian rhythm (Fig. 1C) driven by the altered expression of *arntl*,* npas2*,* nr1d1*,* nr1d2*,* per1*,* pre2*, and *per3*. Altered expression of these genes in the lungs was assessed by quantitative PCR in a 2‐week exposure protocol (Fig. 1D) and confirmed in the 8‐week protocol (same use for microarrays) (Fig. 1E), showing an early and progressive impact of electronic cigarette‐mediated vapors of propylene glycol and glycerol on genes regulating the circadian rhythm (Fig. 1F). Importantly, these changes in pulmonary expression of circadian molecular clock genes took place in the absence of an inflammatory response, with no differences in bronchoalveolar lavage total cell, mononuclear or neutrophil cell numbers, or lung histological changes (Fig. 1G and H).

### Both propylene glycol and glycerol affect the pulmonary expression of circadian molecular clock genes

**Figure 2:**
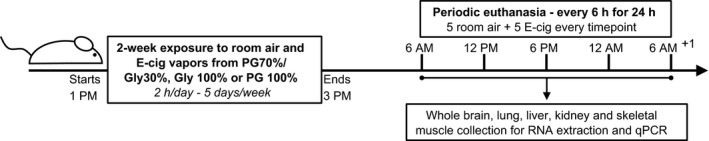
Periodic euthanasia protocol used to assess the impact of electronic cigarette vapors on gene expression.

**Figure 3:**
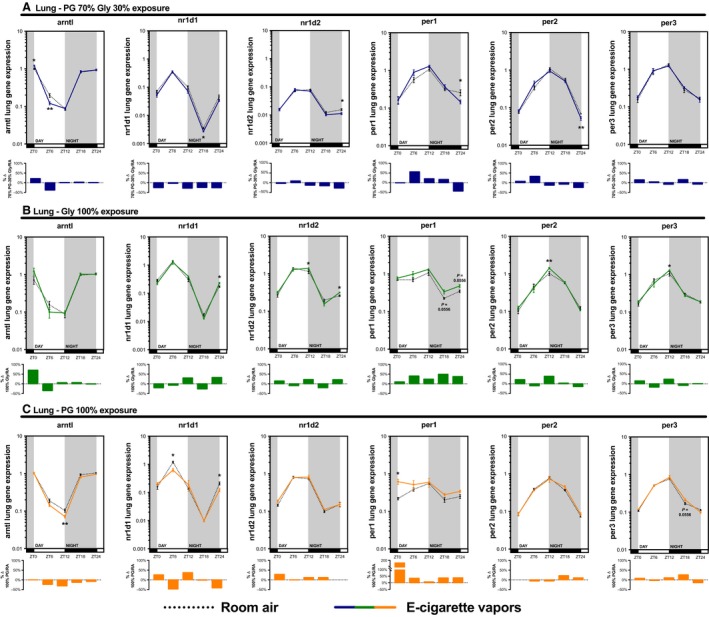
Both propylene glycol and glycerol vapors from electronic cigarette affect the expression of circadian molecular clock genes. BALB/c mice (*n* = 5/group) were exposed to room air (dashed line) or vapors from (A) propylene glycol 70%/glycerol 30% (blue line), (B) glycerol 100% (green line), or (C) propylene glycol 100% (orange line) 2 h/day, 5 days/week for 2 weeks. They were then euthanized according to Figure 2. Lung expression levels of *arntl*,* nr1d1*,* nr1d2*,* per1*,* per2,* and *per3* were assessed by quantitative PCR. Histograms present the percentage of variation between room air and E‐cigarette vapors. Data are presented as mean ± SEM. Mann–Whitney nonparametric test. *: *P* < 0.05; **: *P* < 0.01.

As exposure to e‐cigarette vapors induced variation in expression of clock genes in the lung, we then investigated which of its components, that is, glycerol and/or propylene glycol, was or were responsible. In order to do so, we proceeded to periodical sacrifices of mice exposed to room air, propylene glycol 70%/glycerol 30%, glycerol 100%, or propylene glycol 100% for 2 weeks (Fig. 2). We found that both propylene glycol and glycerol were able to affect the expression of circadian molecular clock genes (Fig. 3). These data show that real‐life variations in e‐liquid solvent mixture, from 100% glycerol to 100% propylene glycol, can alter the pulmonary expression of circadian molecular clock genes.

### Electronic cigarette‐mediated vapors of propylene glycol and glycerol affect the expression of circadian molecular clock genes in the brain, liver, skeletal muscle, and kidney

**Figure 4:**
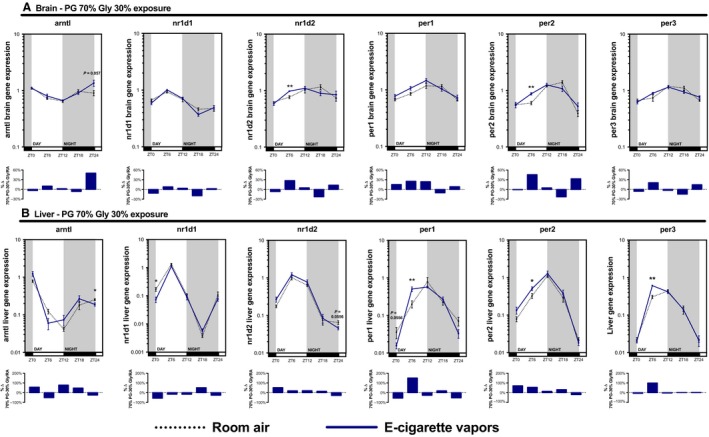
Electronic cigarette vapors impacts the expression of circadian molecular clock genes in the brain and liver. BALB/c mice (*n* = 5/group) were exposed to room air (dashed line) or vapors from propylene glycol 70%/glycerol 30% (blue line) 2 h/day, 5 days/week for 2 weeks. They were then euthanized according to Figure 2. (A) Brain and (B) liver expression levels of *arntl*,* nr1d1*,* nr1d2*,* per1*,* per2,* and *per3* were assessed by quantitative PCR. Data are presented as mean ± SEM. Mann–Whitney nonparametric test. *: *P* < 0.05; **: *P* < 0.01.

**Figure 5:**
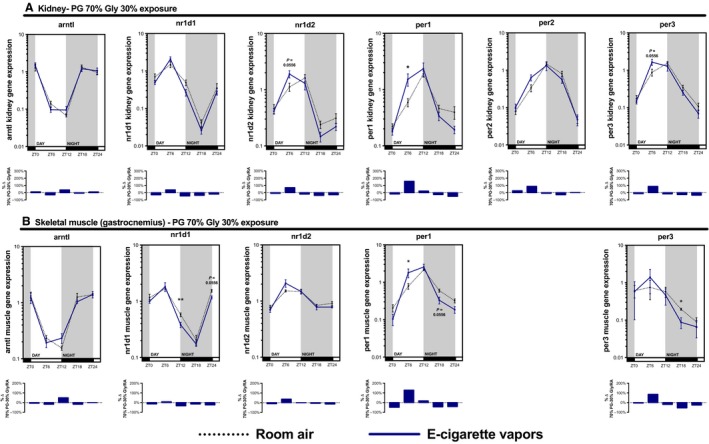
Electronic cigarette vapors impact the expression of circadian molecular clock genes in the kidney and skeletal muscle. BALB/c mice (*n* = 5/group) were exposed to room air (dashed line) or vapors from propylene glycol 70%/glycerol 30% (blue line) 2 h/day, 5 days/week for 2 weeks. They were then euthanized according to Figure 2. (A) Kidney and (B) skeletal muscle (gastrocnemius) expression levels of *arntl*,* nr1d1*,* nr1d2*,* per1*,* per2* (not quantifiable in skeletal muscle), and *per3* were assessed by quantitative PCR. Data are presented as mean ± SEM. Mann–Whitney nonparametric test. *: *P* < 0.05; **: *P* < 0.01.

**Figure 6:**
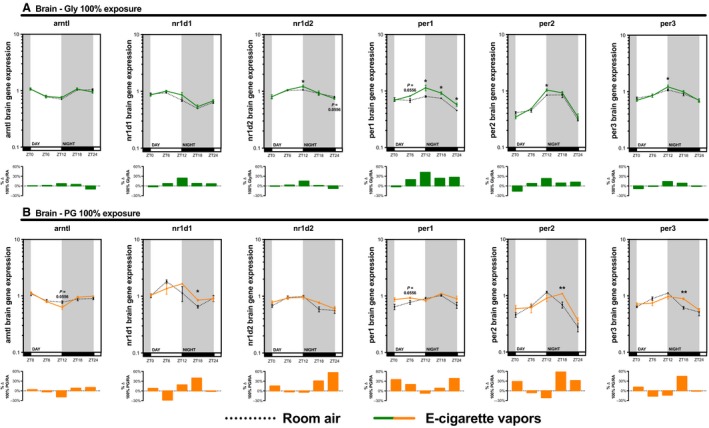
Electronic cigarette vapors from propylene glycol or glycerol alone impact the expression of circadian molecular clock genes in the brain. BALB/c mice (*n* = 5/group) were exposed to room air (dashed line) or vapors from (A) glycerol 100% (green line) or (B) propylene glycol 100% (orange line) 2 h/day, 5 days/week for 2 weeks. They were then euthanized according to Figure 2. Brain expression levels of *arntl*,* nr1d1*,* nr1d2*,* per1*,* per2,* and *per3* were assessed by quantitative PCR. Data are presented as mean ± SEM. Mann–Whitney nonparametric test. *: *P* < 0.05; **: *P* < 0.01.

**Figure 7:**
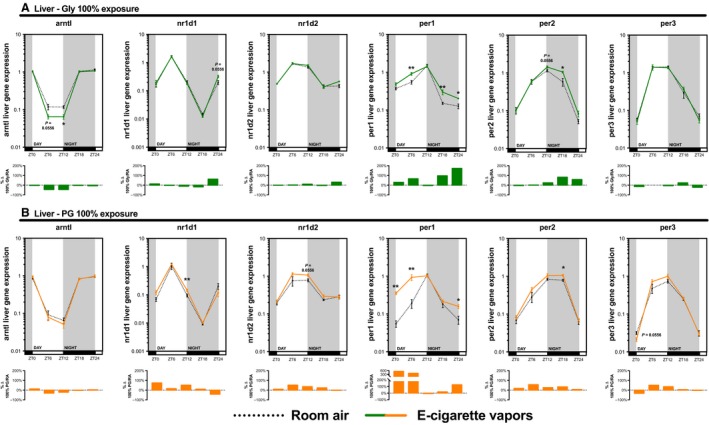
Electronic cigarette vapors from propylene glycol or glycerol alone impact the expression of circadian molecular clock genes in the liver. BALB/c mice (*n* = 5/group) were exposed to room air (dashed line) or vapors from (A) glycerol 100% (green line) or (B) propylene glycol 100% (orange line) 2 h/day, 5 days/week for 2 weeks. They were then euthanized according to Figure 2. Liver expression levels of *arntl*,* nr1d1*,* nr1d2*,* per1*,* per2,* and *per3* were assessed by quantitative PCR. Data are presented as mean ± SEM. Mann–Whitney nonparametric test. *: *P* < 0.05; **: *P* < 0.01.

**Figure 8:**
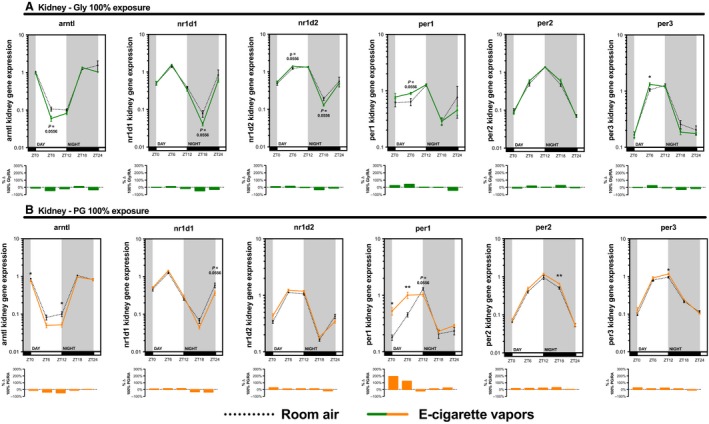
Electronic cigarette vapors from propylene glycol or glycerol alone impact the expression of circadian molecular clock genes in the kidney. BALB/c mice (*n* = 5/group) were exposed to room air (dashed line) or vapors from (A) glycerol 100% (green line) or (B) propylene glycol 100% (orange line) 2 h/day, 5 days/week for 2 weeks. They were then euthanized according to Figure 2. Kidney expression levels of *arntl*,* nr1d1*,* nr1d2*,* per1*,* per2,* and *per3* were assessed by quantitative PCR. Data are presented as mean ± SEM. Mann–Whitney nonparametric test. *: *P* < 0.05; **: *P* < 0.01.

**Figure 9:**
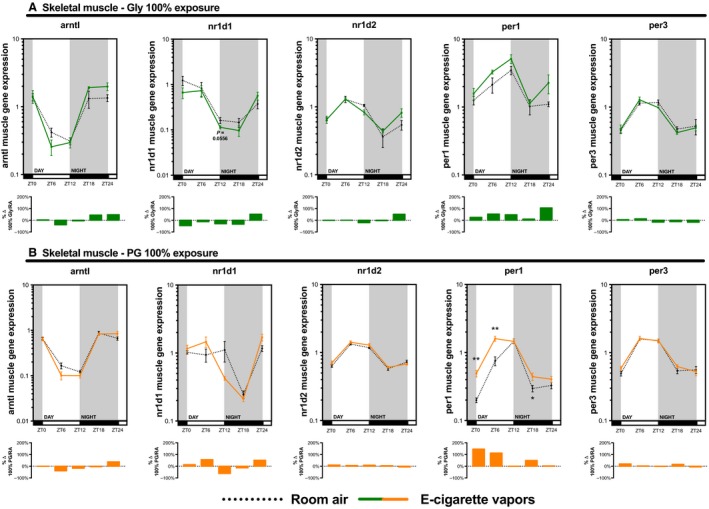
Electronic cigarette vapors from propylene glycol or glycerol alone impact the expression of circadian molecular clock genes in the skeletal muscle. BALB/c mice (*n* = 5/group) were exposed to room air (dashed line) or vapors from (A) glycerol 100% (green line) or (B) propylene glycol 100% (orange line) 2 h/day, 5 days/week for 2 weeks. They were then euthanized according to Figure 2. Skeletal muscle (gastrocnemius) expression levels of *arntl*,* nr1d1*,* nr1d2*,* per1,* and *per3* were assessed by quantitative PCR. Data are presented as mean ± SEM. Mann–Whitney nonparametric test. *: *P* < 0.05; **: *P* < 0.01.

As many inhaled substances, such as cigarette smoke, have systemic effects, we further explored the impact of e‐cigarette vapors on the expression of circadian molecular clock genes in the brain, liver, kidney, and skeletal muscle (gastrocnemius). As for the lungs, these organs were collected periodically from mice exposed to room air, propylene glycol 70%/glycerol 30%, glycerol 100%, or propylene glycol 100% for 2 weeks (Fig. 2). Circadian molecular clock gene expression from the propylene glycol 70%/glycerol 30% exposure is shown in Figure 4 (brain and liver) and Figure 5 (kidney and skeletal muscle) and from glycerol 100% and propylene glycol 100% in Figures 6, 7, 8, 9. In all organs and for many genes, expression appears to be affected at ZT6, and again, changes in gene expression were also observed in organs from animals exposed to glycerol 100% or propylene glycol 100%. Altogether, these data suggest that exposure to e‐cigarette vapors of propylene glycol and/or glycerol can have a systemic impact and affects the expression of circadian molecular clock genes.

### Systemic expression of *hspa1a* and *hspa1b*, widely expressed rhythmic heat shock proteins, is affected by electronic cigarette vapors

**Figure 10:**
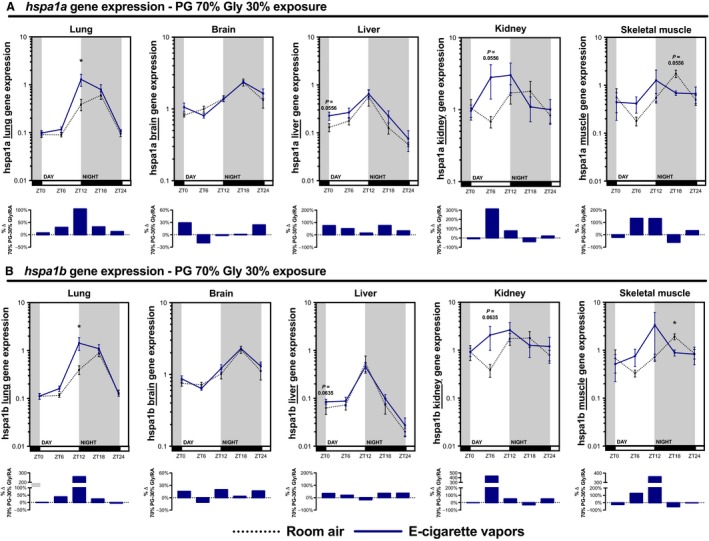
Electronic cigarette vapors impact gene expression of *hspa1a* and *hspa1b* in various organs. BALB/c mice (*n* = 5/group) were exposed to room air (dashed line) or vapors from propylene glycol 70%/glycerol 30% (blue line) 2 h/day, 5 days/week for 2 weeks. They were then euthanized according to Figure 2. Expression levels of (A) *hspa1a* and (B) *hspa1b* were assessed in the lungs, brain, liver, kidney, and skeletal muscle. Data are presented as mean ± SEM. Mann–Whitney nonparametric test. *: *P* < 0.05.

*Hspa1a* and *hspa1b*, genes coding for Hsp70 members 1a and 1b, are widely expressed and depict circadian rhythmicity (Sandström et al. 2009; Ackermann et al. 2013). Lung expression of these two genes was upregulated by e‐cigarette in the initial microarray (Fig. 1B). To investigate if circadian expression of *hspa1a* and *hspa1b* was affected by e‐cigarette vapors, we measured mRNA levels in the lungs, brain, liver, kidney, and skeletal muscle. We found that *hspa1a* and *hspa1b* expression was altered in the lungs, liver, kidney, and skeletal muscle but not in the brain (Fig. 10). This suggests that expression of genes depicting circadian rhythmicity, such as *hspa1a* and *hspa1b*, could be affected by exposure to e‐cigarette vapors.

## Discussion

The impacts of electronic cigarette vapors on the lungs are yet to be fully understood. The principal constituents of e‐liquids, namely, propylene glycol and glycerol, are less studied than nicotine and flavoring agents and widely responsible for the perceived safety of electronic cigarette (Camenga et al. 2015; Farsalinos et al. 2015; Hummel et al. 2015). To address the impact of inhaling vapors of these two specific compounds, we designed a mouse whole‐body exposure system. Lung expression microarray analysis revealed that vapors from a mixture of propylene glycol and glycerol modulated the expression of circadian molecular clock genes, despite no apparent sign of lung inflammation. Both propylene glycol and glycerol alone were able to cause this phenomenon. Finally, altered expression of circadian molecular clock genes was also observed in the brain, liver, kidney, and skeletal muscle. Altogether, these data suggest that inhaling vapors of propylene glycol and glycerol generated by an electronic cigarette can affect the expression of circadian molecular clock genes in a systemic fashion.

We showed that inhalation of propylene glycol and glycerol vapors can, together or separately, impact the expression of circadian molecular clock genes. We currently do not know the mechanisms behind this phenomenon. However, we can postulate some potential mechanisms. Glycerol can be used as a source of energy. Glycerol metabolism and release in the circulation is known to be circadian (Shostak et al. 2013). Both propylene glycol and glycerol can be metabolized into lactate (Jensen et al. 2017). Production of lactate also shows circadian rhythmicity in various organs (Isobe et al. 2011a, 2011b). Glycerol and lactate sensing mechanisms could interact with the regulatory elements of the circadian molecular clock to affect the peripheral clocks at specific times. Due to the extent of the phenomenon, it is more likely that glycerol and propylene glycol‐mediated transcriptional alterations of circadian molecular clock genes is related to metabolic sensing caused by a very specific, concentrated, and defined source of energy/metabolites entering the body from an unusual place. Further investigations are required to decipher the underlying biology.

Expression of *hspa1a* and *hspa1b* is altered by inhalation of propylene glycol and glycerol vapors in various organs. While we cannot say if the modulation of these specific genes is of biological relevance, this shows that genes not involved in controlling the circadian molecular clock but depicting circadian rhythmicity can be affected by propylene glycol and glycerol vapors inhalation in multiple organs. This is of importance as most biological functions are controlled or at least affected by the circadian rhythm. Glucose metabolism (Panda et al. 2002; So et al. 2009; Bass and Takahashi 2010), immune and inflammatory responses (Oishi et al. 2006; Gibbs et al. 2009, 2014; Keller et al. 2009; Castanon‐Cervantes et al. 2010; Ackermann et al. 2012; Scheiermann et al. 2012; Bellet et al. 2013; Sato et al. 2014), lipolysis (Turek et al. 2005; Chaput et al. 2006; Kohsaka et al. 2007; Mukherji et al. 2015), only to name a few, are crucial biological functions affected by the circadian rhythm. Therefore, global deregulation in circadian rhythmicity, even if mild or only at specific times of the day/night could have biological repercussions.

Disruption in circadian rhythmicity can cause numerous biological alterations and exacerbate pathologies. In the lungs, disrupted circadian rhythmicity can exacerbate the response to bacterial agents by modulating the amplitude of the response to endogenous cortisol (Gibbs et al. 2009, 2014). In the periphery, Clock‐deficient animals are dyslipidemic and prone to weight gain (Turek et al. 2005). Furthermore, deletion of *clock* or *bmal1* in mice triggers diabetes mellitus, characterized by impaired glucose tolerance and decreased insulin secretion (Marcheva et al. 2010). As of now, it is unlikely that electronic cigarette use would cause severe pathologies over time, even though we cannot rule it out. However, it is very likely that its effects on the circadian molecular clock could amplify, accelerate, or change the course of pathological processes. Animal models will be instrumental in exploring these potential effects.

This study has limitations. First, whole‐body exposure does not reflect exactly how humans are exposed to electronic cigarette vapors. The dose is also difficult to estimate. Keeping in mind that e‐cigarette users do not all inhale the same way or use the same dosage, we are confident that this exposure model provides a worthy proof‐of‐concept for e‐cigarette‐mediated pulmonary and systemic phenomenon. Our exposure system is similar, in principle, to most tobacco cigarette smoke exposure systems, which have been and still are instrumental in deciphering the mechanisms behind its pulmonary and systemic consequences (General A. R. O. T. S). Due to limitations of our exposure system and the number of animals that can be exposed at the same time, resolution of the circadian rhythm was limited to 6 h and to one cycle of 24 h. Therefore, it remains unclear if PG and Gly affect the period, amplitude, phase, or mesor of clock gene expression. Finally, mice are nocturnal while human are diurnal and we cannot exclude that this did not impact the observed phenomenon.

This study reveals that the major solvents used in electronic cigarettes have unsuspected effects on the expression of circadian molecular clock genes that are not to be taken lightly. Most importantly, these effects are not mediated by nicotine or flavors but by propylene glycol and glycerol, two compounds with next to no toxicity when ingested and at the very source of the perceived ‘safety’ of electronic cigarette. As this habit keeps increasing in popularity, a better understanding of the biological effects of propylene glycol and glycerol inhalation as well as the potential interactions with other pulmonary or systemic conditions or pathologies is required.

## Conflict of Interest

The authors have declared that no conflict of interest exists.
